# Characterization of Plastidial and Nuclear SSR Markers for Understanding Invasion Histories and Genetic Diversity of *Schinus molle* L.

**DOI:** 10.3390/biology7030043

**Published:** 2018-08-10

**Authors:** Rafael Plá Matielo Lemos, Cristiane Barbosa D’Oliveira Matielo, Dalvan Carlos Beise, Vanessa Gonçalves da Rosa, Deise Schröder Sarzi, Luiz Fernando Würdig Roesch, Valdir Marcos Stefenon

**Affiliations:** 1 Núcleo de Ecologia Molecular e Micropropagação de Plantas, Universidade Federal do Pampa, São Gabriel ZIP 97307-020, Brazil; rafael.matielo@unipampa.edu.br (R.P.M.L.); crixdoliveira@gmail.com (C.B.D.M.); dalvanbio@gmail.com (D.C.B.); vaneessagr@hotmail.com (V.G.d.R.); 2 Instituto de Bioquímica Médica Leopoldo de Meis—CCS, Universidade Federal do Rio de Janeiro, Rio de Janeiro 21941-902, Brazil; deisesarzi@hotmail.com; 3 Centro Interdisciplinar de Pesquisas em Biotecnologia—CIP-Biotec, Campus São Gabriel, Universidade Federal do Pampa, São Gabriel ZIP 97307-020, Brazil; luizroesch@unipampa.edu.br

**Keywords:** invasive species, genetic diversity, genetic resources, molecular markers

## Abstract

Invasive plant species are expected to display high dispersal capacity but low levels of genetic diversity due to the founder effect occurring at each invasion episode. Understanding the history of invasions and the levels of genetic diversity of such species is an important task for planning management and monitoring strategy for these events. Peruvian Peppertree (*Schinus molle* L.) is a pioneer tree species native from South America which was introduced in North America, Europe and Africa, becoming a threat to these non-native habitats. In this study, we report the discovery and characterization of 17 plastidial (ptSSR) and seven nuclear (nSSR) markers for *S. molle* based on low-coverage whole-genome sequencing data acquired through next-generation sequencing. The markers were tested in 56 individuals from two natural populations sampled in the Brazilian Caatinga and Pampa biomes. All loci are moderately to highly polymorphic and revealed to be suitable for genetic monitoring of new invasions, for understanding the history of old invasions, as well as for genetic studies of native populations in their natural occurrence range and of orchards established with commercial purposes.

## 1. Introduction

**Figure 1:**
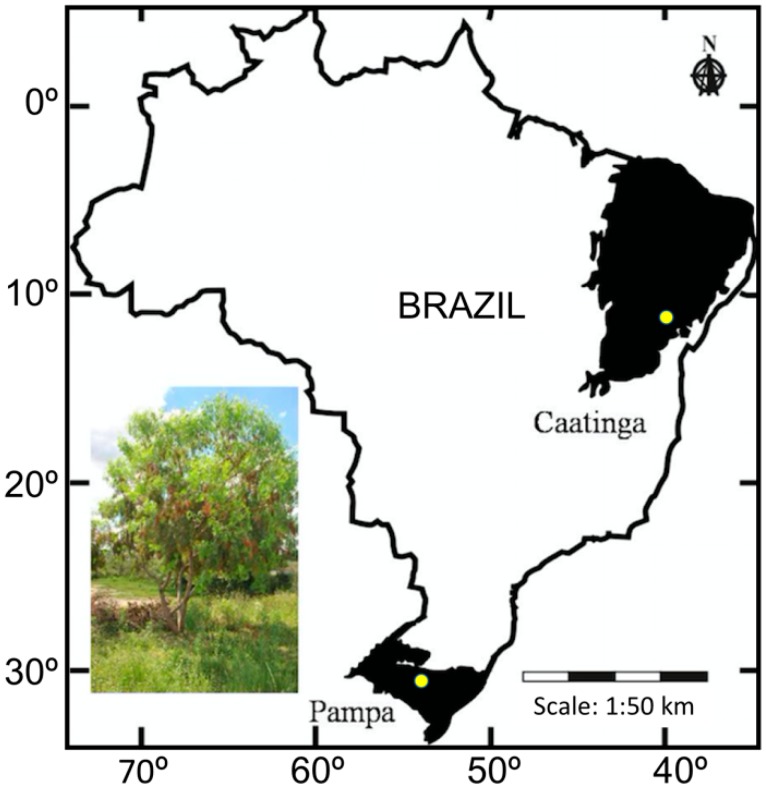
Location of the Pampa and Caatinga biomes in Brazil, where samples of *Schinus molle* were collected for this study. Dots within the biomes represent the location of each sampled population. Insert: fruiting adult tree of *S. molle*, within the Pampa biome.

The Peruvian Peppertree (*Schinus molle* L., Anacardiaceae; Figure 1) is a pioneer tree species native from South America and a threat to many environments around the world. This species can flower at any time due to its quick phenological cycle, which makes their seeds available for dispersal throughout the year [1,2]. Claimed to be an alien and invasive species, *S. molle* has plasticity and a natural ability to establish and survive in many environments. Well-known for its application in popular medicine [3], by the pharmaceutical uses of its essential oils [4], and by the production of the spicy pink pepper [5], *S. molle* has an important role in the medical, pharmaceutical and food industries. On the other hand, the reports of this species’ invasion in California [6], Israel [7], Hawaii [8], South Africa [9], Mexico [10] and Italy [11] highlight the urgent need for tools to manage and control its dispersion capacity and large ability to establish in new environments. This scenario may become worse under the current climatic changes, as demonstrated by ecological niche modelling (ENM) of *S. molle* distribution. A recent ENM study of *S. molle* potential distribution areas in Southern South America showed that there is a high probability of this species expanding over the Atlantic Forest [12]. Additionally, the risk of invasion of other of the world’s biodiversity hotspots by this species is highly likely, due to its high adaptability into several environments.

Invasive species tend to present low genetic diversity due to the founder effect, i.e., the reduction in genetic diversity seen when a small subset of individuals splits from a larger and more diverse population, forming the basis of a new one [13]. Accordingly, low genetic diversity was observed in nine populations of *S. molle* studied in its natural range of occurrence in southern Brazil, based on dominant amplified fragments length polymorphism (AFLP) markers [14].

Mainly due to their locus-specific nature, co-dominance and high polymorphism, simple sequence repeats (SSR) markers have been used effectively for assessing genetic diversity, population differentiation, intra-populational genetic structure, interpopulation gene flow, and fitness of natural populations (e.g., [15,16]). SSR markers can be characterized in both organellar or nuclear genomes. Organellar markers (plastidial and mitochondrial) have been widely used in phylogeographic studies, mostly due to the absence of recombination and the mainly uniparental (maternal) heritability. Neutral nuclear SSR markers (SSR loci found within non-expressed regions of the genome) are important to measure neutral DNA variation and are quite useful in populational studies.

There is a large number of SSR loci spread out all over the eukaryotes genomes, in coding and non-coding nuclear and organellar DNA [17]. Next-generation sequencing (NGS) platforms enable quick and cost-effective studies on small genomes, generating data of huge importance for biotechnological exploitation, conservation and breeding of non-model tree species, including the discovery of SSR markers [17]. Also, the use of molecular techniques has proved to be successful in reconstructing the history of invasions and evolutionary changes that may have occurred since the introduction of the species into non-natural environments [18].

Aiming to generate molecular markers for the close characterization of genetic diversity and structure, as well as the evolutive patterns and history of natural and invasive populations of *S. molle*, we employed NGS for the development of organellar and nuclear SSR markers. Here we report the development and characterization of 24 SSR markers that presented moderate to high polymorphism when employed for genotyping adult individuals from two natural populations of *S. molle*. In addition, we discuss the outcomes of individual and combined use of the organellar (plastidial) and nuclear markers characterized.

## 2. Materials and Methods

### 2.1. Sample Strategies and DNA Extraction

Two natural populations of *Schinus molle* were mapped and collected in Pampa and Caatinga Biomes in Brazil (Figure 1). Twenty-seven individuals of population Pampa were sampled in the municipality of São Gabriel, Rio Grande do Sul State (30°21′ S, 54°18′ W), and 29 individuals were sampled in the Caatinga, Feira de Santana municipality, Bahia State (12°14′ S, 38°57′ W). Considering the commonly observed polymorphism of SSR loci and the literature related to characterization of SSR markers, this number of samples, equally distributed in two populations growing in different biomes enables the characterization of the polymorphism and usefulness of the discovered SSR loci at both, inter- and intrapopulation levels. Fresh leaves from each tree were sampled and individually conditioned in plastic bags with silica gel until the DNA extraction.

Total DNA was extracted using the protocol CTAB 2% [19] slightly modified. Quality of the isolated DNA was determined using a NanoVue™ spectrophotometer (GE Healthcare, Chicago, IL, USA). DNA was purified twice with the Agencourt^®^ AMPure^®^ XP Reagent (Beckman Coulter, Brea, CA, USA) and the final concentration of the DNA was quantified by using the Qubit Fluorometer kit (Invitrogen, Carlsbad, CA, USA) following the manufacturer’s recommendations. The DNA samples were stored at −20 °C until use.

### 2.2. NGS Sequencing and de novo Assembly

A single plant of the population Pampa was selected for the NGS sequencing. An herbarium voucher of this sample was deposited in the Bruno Edgar Irgang Herbarium (HBEI) of the Federal University of Pampa under the number 1572. Total DNA from this plant was used for library preparation with Ion OneTouch™ 2 System using the Ion PGM™ Template OT2 400 Kit (Thermo Fisher Scientific, Waltham, MA, USA). The sequencing was performed using Ion PGM™ Sequencing 400 kit on the Ion PGM™ System with an Ion 318™ Chip v2.

The FastQ file exported from the Ion PGM^TM^ System was evaluated for the K-mer number by KmerGenie software [20], and the *de novo* assembly was performed using the Velvet software [21].

### 2.3. Discovery and Characterization of SSR Markers

The software SSRLocator [22] was used to find di- and tri-nucleotide repeat motifs in the obtained contigs of the *S. molle* sample. The default parameters of SSRLocator were employed to identify SSR loci with a minimum of four and three repetitions for dimer and trimer motifs respectively. Primers for the identified SSR loci were designed using the Primer3 software [23], searching for alleles with sizes ranging from 90 to 500 bp. All contigs containing SSR loci were deposited in the GenBank (ID numbers are listed in Table 1).

Genomic origin of each SSR locus was identified through a BLAST search on the NCBI’s website. For this search, each contig containing the SSR locus was compared to sequences of Viridaeplantae deposited in the GenBank using the BLASTn tool and the MegaBLAST strategy. The bit-score and the percentage of identity of each contig to the subject of the BLAST analysis were used as conditions for determining the genomic origin of each SSR locus. According to the outcome of this search, SSR loci were classified as (i) a plastidial SSR locus (ptSSR) when the sequence was located within the plastome, or (ii) a nuclear neutral SSR locus (nSSR) when the contig failed to match with any sequence within the GenBank database. Such an outcome was interpreted as the contig being located in non-expressed regions within the nuclear genome.

An *in silico* amplification was performed to test the potentially amplifiable SSR loci using the software SPCR [24]. With this strategy, we were able to identify primer pairs that were potentially amplifying a single locus in *S. molle* genome within the expected size range and discard primer pairs that were generating multi-loci amplifications and unfeasible band patterns. SSR loci presenting single locus amplifications in the *in silico* analyses were characterized in the 56 individuals from the sampled natural populations.

Selected SSR markers were amplified through PCR in a reaction mix with a final volume of 12.5 μL, which was prepared using an Eppendorf epMotion^®^ 5070 (Eppendorf AG, Hamburg, Germany) containing about 50 ng of DNA, 0.25 μM of buffer, 0.5 μM of MgCl_2_, 1 U of Taq DNA-Polymerase (Invitrogen^®^), 0.05 μM of each dNTP, 0.125 μM of forward primer, 0.125 μM of reverse primer, and 0.125 μM of the M-13 primer (5′-TGTAAAACGACGGCCAGT-3′) labelled with AlexaFluor 680 (Invitrogen^®^) fluorescence. The forward primer of each pair holds an M-13 tail, complementary to the fluorescent M13-primer (Table 1). Amplifications were carried out with an initial step of 94 °C for 3 min, a denaturing period of 94 °C for 45 s, annealing temperature ranging from 47 °C to 55 °C (see Table 1) for 30 s and extension at 72 °C for 1 min, for a total of 34 cycles, with a final extension step of 72 °C for 10 min in a BIO-RAD C1000 Touch™ Thermal Cycler (BioRad Co., Hercules, CA, USA).

SSR amplifications were electrophoretically separated on 6% polyacrylamide gels using a Li-Cor 4300 S automated DNA Sequencer^®^ ((LiCor Inc., Lincoln, NB, USA) and automatically scored and analyzed in SAGA^GT^ Software^®^ (LiCor Inc.). An additional visual check of each scored gel was made in order to correct possible mismarked bands.

Total number of alleles (*A*), effective number of alleles (*A_e_*), observed heterozygosity (*H_O_*), expected heterozygosity (*H_E_*), within population fixation index [*F_IS_* = (*H_E_* − *H_O_*)/*H_E_*], and deviation from Hardy-Weinberg equilibrium (HWE) were estimated for each locus in each population and overall. Differentiation between populations was estimated using the AMOVA approach. All estimations were performed using the software GenAlEx 6.5 [25,26].

Linkage disequilibrium between pair of SSR loci was estimated using GenePop on web version 4.6 (http://genepop.curtin.edu.au) [27,28]. The Markov chain parameters used in the analysis were 1000 dememorization steps, 100 batches and 1000 iterations per batch.

## 3. Results

### 3.1. Sequencing Output and SSR Discovery

The low-coverage genome sequencing generated around 578 Mb of sequences that were used in the *de novo* assembly. A total of 1.267.774 bp with a GC content of 37% were obtained, corresponding to about 0.3X coverage of the *S. molle* genome, the estimated size of which is of about 410 Mbp [29]. Despite the low coverage of the genome, the sequencing enabled the discovery of more than 200 SSR sequences, 69 of which enabled the design of primers with a GC content of between 40 and 60%, closely matched annealing temperatures for forward and reverse primers, absence of undesirable secondary structures produced by intra- or intermolecular interactions (hairpins, self-dimer, cross-dimer) and generated a putative allele within the size range of 90 to 500 bp. These 69 loci were then further screened through *in silico* tests for amplification.

After the *in silico* test for amplification, 30 out of the 69 prospective SSR loci were considered optimal, presenting the amplification of a single locus within the expected range and were considered to be putative informative SSR markers. These 30 SSR loci were then characterized for polymorphism in two natural populations of *S. molle*.

### 3.2. Characterization of SSR Markers

Out of the 30 SSR markers tested in natural population samples, 24 presented amplifications within the expected range, while five markers failed at amplifying, even after replications of the PCR procedures. One SSR locus located within the mitochondrial genome of *S. molle* was excluded from further analysis. Of these 24 markers, 17 were located within the plastome (ptSSR markers), and seven in non-expressed regions within the nuclear genome (nSSR markers). The contigs containing the characterized SSR loci had a mean size of 540 bp and presented identity ranging from 94 to 100% of the contig (including the primers, the flanking regions and the SSR region) to the subject sequences of the BLAST analysis. All contigs presenting such high similarity to the subject sequences were located within the plastidial genome and were classified as plastidial SSR markers (ptSSR; Table 1). Seven contigs failed to match with sequences deposited in the GenBank. Because non-expressed nuclear intergenic regions are usually not deposited in this databank, we considered these seven SSR loci to be located within non-expressed regions of the nuclear genome of *S. molle* and classified them as neutral nuclear SSR markers (nSSR; Table 1). The repeat motifs, forward and reverse primers, annealing temperature, percent of identity of the contigs to the subject sequence and the GenBank ID number of the primers of the 24 characterized SSR markers are listed in Table 1.

Moderate to high polymorphism was found in all 24 SSR markers tested in both populations (Table 2 and Supplementary Table S1). A total of 242 alleles were observed, ranging from 6 to 22 alleles per locus (Supplementary Table S1), with a mean *A* of 10.08 (Table 2); while the mean effective number of alleles per locus was *A_e_* = 6.03, ranging from 2.52 to 10.56 (Supplementary Table S1). Expected and observed heterozygosities ranged from 0.60 to 0.90 (mean *H_E_* = 0.81) and 0.00 to 1.00 (mean *H_O_* = 0.64), respectively. The within-population fixation index (*F_IS_*) ranged from −0.22 to 1.00 (mean *F_IS_* = 0.21). Except for the loci Smolle09 and Smolle25 in the population Pampa, a significant deviation of HWE (*p* < 0.05) was observed in all loci in the overall analysis (Supplementary Table S1).

When evaluated individually in the population Caatinga, plastidial markers revealed lower estimations of genetic diversity (*A*, *A_e_*, *H_O_* and *H_E_*) than neutral markers (Table 3). In the population Pampa, lower estimations were observed for the neutral nSSRs for *H_O_* and *H_E_* (Table 3). Congruently, the estimation of the within-population fixation index (*F_IS_*) was higher for plastidial markers in the population Caatinga, while it was higher for nSSR markers in the population Pampa. When the different markers are combined, estimations of genetic diversity present low variation within each population (Table 3).

The AMOVA approach revealed statistically significant (*p* < 0.001) differentiation between populations *F_ST_* = 0.13 for all markers combined, *F_ST_* = 0.14 for ptSSRs, *F_ST_* = 0.11 for nSSRs, indicating a higher structure related to markers found in plastidial regions.

Estimations of linkage disequilibrium (LD) revealed 55 pairs of loci with significant (*p* < 0.05) LD in the population Pampa and 71 in the population Caatinga (Supplementary Table S2). Of these, 11 pairs of loci revealed significant LD in both populations (Table 4), suggesting physical ligation instead of inbreeding as the cause of this departure. Indeed, four out of the 11 pairs show ligation between plastidial markers and present physical ligation in the circular chromosome of the chloroplast. However, seven pairs are formed by one plastidial and one nuclear marker, suggesting the effect of inbreeding, because physical ligation between plastidial and nuclear loci is not possible.

## 4. Discussion

In this study, we identified and characterized 17 ptSSR and seven nSSR markers for the invasive species *S. molle* based on low-coverage whole-genome sequencing. Non-model tree species usually lack information about genome sequences, making the development of molecular markers based on the knowledge of these data difficult and expensive. However, with the advance of NGS technologies in recent years, low-coverage whole-genome sequencing has been increasingly used for the development of SSR markers for such species. This approach was successfully used for developing SSR markers for several tree species with recognized economic importance [30,31,32] and, to our knowledge, this is the first report of using this approach for developing markers for an invasive tree species. Low coverage of the whole genome is a useful technique for sketching the genomic composition of a species [32], and even with particularly low-coverage sequencing, bioinformatics methodologies can generate extensive information about functional and repetitive elements, providing useful novel genomic resources [33].

The 24 SSR markers developed for *S. molle* presented moderate to high allelic diversity and heterozygosity in two natural populations, suggesting high usefulness for the characterization of genetic diversity and history in invaded areas. The two populations used in this study represent divergent biomes in Brazil and, therefore, a significant difference is expected between them, related to adaptation to the edafoclimatic conditions. The pairwise population differentiation estimated based on allele frequencies using the AMOVA approach was significant for each set of markers (ptSSRs, nSSRs) evaluated independently or combined. The slightly higher differentiation estimated with ptSSRs in comparison to nSSRs may be the effect of a selective sweep over some plastidial genes, affecting all SSR loci linked in the single plastidial chromosome in a genetic hitchhiking process. In fact, most of ptSSR loci revealed private alleles in both Pampa and Caatinga populations (Supplementary Table S3), supporting the existence of selection. Such private alleles may be very useful markers for determining the origin of individuals, and to reconstruct the history of invasion in recently occupied areas or even old invasions based on the frequency of particular alleles. These ptSSR markers may also be useful for differentiating populations growing under different selective pressure and to identify the origin of plants invading a determined environment.

In addition, assuming strict uniparental inheritance of the chloroplast in *S. molle*, the pollen-to-seed migration ratio (*m_p_*/*m_s_*) may be assessed from the plastidial and nuclear markers characterized in this study, using the estimations of *F_IS_* obtained from nSSRs and *F_ST_* obtained from each marker class individually [34]. Using the *F_IS_* and *F_ST_* estimations from this study, *m_p_*/*m_s_* equaled 4.14 (see Supplementary file S4 for details about *m_p_*/*m_s_* computation), suggesting that seed dispersal plays a more important role than pollen in the total gene flow [34]. However, given the very large geographical distance splitting the Pampa and Caatinga populations, this estimation has no biological meaning, being used here merely for illustrative purposes.

The comparison between the two sets of markers did not diverge significantly in the patterns of diversity within each population. Recognizably low diversity and high fixation index were observed in population Caatinga, which experiences harder conditions in comparison to the population Pampa. Caatinga is a warm and dry biome with very specific and endemic xeric vegetal components [35,36], while the Pampa is a savanna-like environment with very few forest patches along its extension and a somewhat moist climate with four well characterized seasons [37].

Nuclear neutral SSRs are expected to present high levels of polymorphism due to their occurrence in the less conserved genomic regions. The ptSSR loci characterized for *S. molle* in this study presented basically the same estimations of nSSR loci, suggesting that despite the likely genetic hitchhiking process causing higher genetic differentiation at ptSSR markers, the selection is not strong when comparing the populations Caatinga and Pampa.

The elevated number of pairs of loci with significant LD observed in both populations is likely an effect of their isolation and small size, as also observed in the positive significant fixation index and the deviation from Hardy-Weinberg equilibrium in the ptSSR and nSSR markers for both populations. The ligation between pairs of ptSSR markers is not surprising, since they are located in the same circular plastidial chromosome. However, the significant LD for pairs of loci composed by plastidial and nuclear regions does not represent physical linkage, since they are located in different genomes.

## 5. Conclusions

Low-coverage whole-genome sequencing was employed in this study, with the aim of developing SSR markers for genetic studies of *S. molle*. The 24 SSR markers characterized here are moderately to highly variable and are useful for studies concerning the levels of diversity, the genetic differentiation, patterns of seed/pollen dispersal and the history of invasive populations of *S. molle* across the world, as well as for genetic studies of native populations in their natural occurrence range and of orchards established for commercial purposes.

Either employed independently or combined, both set of markers characterized in this study are very useful. Although linked in the single plastidial chromosome, ptSSR markers are highly informative and have been successfully used in phylogeographic studies, being the marker employed in 42% of the studies on this topic performed in the last decade [38], and also useful in population genetic diversity and structure analyses [39]. The neutral nuclear SSR markers, in turn, are quite useful in population genetic studies, since they are primarily not subjected to evolutionary forces and are likely to be distributed across the different chromosomes of *S. molle*, since no linkage disequilibrium between pairs of nSSR loci was observed.

## Figures and Tables

**Table 1:** List of 24 SSR markers characterized for *Schinus molle*, including primer sequences (forward and reverse), repeat motif, length of the sequenced fragment, annealing temperature (Ta), genome region, GenBank accession number (GenBank ID), the bit-score and the identity of the contig to the subject sequence in the BLAST analysis.

Locus	Primer Sequence (5′→3′) ^a^	Rep Motif	Prod. Size	Ta (°C)	Genome Region ^b^	GenBank ID	Bit-Score	Ident.
Smolle03	AAGTTTTATTTTCCCAGAAT AATCATAGGTTCTTCTCTCC	(TTC)_3_	169	51	ptSSR	MH536214	1050	99%
Smolle04	CTCCTAGGGATAAGAGACAT GAATAATTGTTGGAGACTCA	(AG)_4_	206	49	ptSSR	MH536215	941	99%
Smolle05	CGTAGACCAAATGATACAAT TTATTTCTCATCAAACGAAT	(AGA)_3_	261	47	ptSSR	MH536216	941	99%
Smolle06	GGTCCATGAATCTAAGAAAT TTGAAATGAAATCTTTAGGA	(TC)_4_	140	47	ptSSR	MH536217	1014	100%
Smolle07	GAGTTGAAAATAAGCGTAGA TCTGGCTACTAAGATGTTTC	(CTG)_3_	117	51	ptSSR	MH536218	1022	99%
Smolle08	ATTTGTTATCTCATGTTTGC ACACATTGTCTAACCAAATC	(GA)_4_	275	49	ptSSR	MH536219	970	99%
Smolle09	CCCATTAACATTTTAGAAGA GCTAAAGTTGCAAAAATAAG	(GTT)_3_	188	47	ptSSR	MH536220	625	98%
Smolle10	TAGTTCATCCTATTGGCTC GAAACGAATTTTCATTTTTA	(TAA)_3_	234	47	ptSSR	MH536221	492	95%
Smolle11	AGAGGAGTAGTTATGAACCC TCACTATATTTATTCCTTTTTCT	(GAA)_3_	140	51	ptSSR	MH536222	837	99%
Smolle12	CCACTAGAGATCAGAAATTG AATTGAGACGGTATTTTGTA	(AAG)_3_	128	47	ptSSR	MH536223	580	99%
Smolle13	CTGTGTTTTTGGTAACAGTC GGTGGGTAGGTAGAGAATAC	(CT)_4_	148	55	ptSSR	MH536224	1369	99%
Smolle14	AGTTTCTTTTTACACATCCA AAGAAGATCCATTTTGAGTT	(AT)_4_	161	47	ptSSR	MH536225	523	96%
Smolle15	GTACAAATAAGAATCCCCTT AGATCTTGTAGCACTTACCA	(TTC)_3_	268	51	ptSSR	MH536226	1387	99%
Smolle16	GCAGATTCATCTAATTATGG AAGTTATAAGTTGTGAAGCG	(TCT)_3_	193	49	ptSSR	MH536227	588	98%
Smolle19	GTCAACTAAGGGGATAAGAT ATCCAATATCAATAAACCAA	(AT)_6_	159	47	ptSSR	MH536230	1061	97%
Smolle22	CTATAGTGGCTAGGGTGAG AATACCTTCCTCTGTCATCT	(AG)_4_	172	51	ptSSR	MH536232	483	94%
Smolle28	ATTTGTGCTCAATTTTCATA ACACATTGTCTAACCAAATC	(GA)_4_	124	49	ptSSR	MH536237	494	96%
Smolle17	AGAATTCTCTACCATTCTCC ACAAAAATCACATGAAAATC	(TTC)_3_	203	47	nSSR	MH536228	-	-
Smolle18	TCTAGTACCAGAATCTTTGC AGGAGGTAAATCCAACTATC	(CT)_5_	127	51	nSSR	MH536229	-	-
Smolle21	GTGTTTCGTTAAGACAAAAG GTGAGAAACGAATAAAGAAA	(TG)_5_	113	47	nSSR	MH536231	-	-
Smolle23	GAAGATAAGTTCATACCCCT TTCATTAATTGGCTCTAATC	(CTT)_3_	202	47	nSSR	MH536233	-	-
Smolle24	AGATTTCCCGAACTATTATT TGTTCAAGGAATAAAGGTAA	(TGA)_3_	226	47	nSSR	MH536234	-	-
Smolle25	TGCACCTTATATGAAAGACT ACCATCACTACAGCTCATAC	(GAA)_3_	145	53	nSSR	MH536235	-	-
Smolle27	AGTCAATGAAGTTTTCACAG TGAGAACTCAAGATGCTATT	(CT)_4_	224	49	nSSR	MH536236	-	-

^a^ The forward primers contained the M-13 tail (5′-TGT AAA ACG ACG GCC AGT-3′) at the 5′end. ^b^ ptSSR = plastidial markers; nSSR = nuclear neutral markers.

**Table 2:** Multilocus estimations of genetic diversity (*A*, *Ae*, *H_O_* and *H_E_*) and within population fixation index (*F_IS_*). The mean number of samples (*N*) is given. Values are the average across 24 SSR loci.

	*N*	*A*	*A_e_*	*H_O_*	*H_E_*	*F_IS_*
Pampa	23.62	5.54	3.56	0.64	0.68	0.08
Caatinga	24.75	7.33	5.15	0.62	0.72	0.20
Overall	48.37	10.08	6.03	0.64	0.81	0.21

**Table 3:** Mean values of genetic parameters estimated for *Schinus molle* based on the 24 SSR markers characterized in this study. Data are presented independently for each genomic region, plastidial (ptSSRs) and neutral nuclear (nSSRs), for each population. Estimations include the number of samples (*N*), number of alleles per locus (*A*), effective allele number (*A_e_*), observed (*H_O_*) and expected (*H_E_*) heterozygosities, and within-population fixation index (*F_IS_*).

	ptSSRs		nSSRs	
	Caatinga	Pampa	Caatinga	Pampa
*N*	25.17	24.82	23.71	20.71
*A*	6.70	5.70	8.85	5.14
*A_e_*	4.67	3.55	6.32	3.57
*H_o_*	0.57	0.67	0.75	0.56
*H_e_*	0.68	0.68	0.82	0.66
*F_IS_*	0.25	0.03	0.10	0.22

**Table 4:** Pairs of loci with statistically significant (*p* < 0.05) estimations of linkage disequilibrium (LD) in both populations. Supplementary Table S2 presents the list of all pairs of loci with significant LD. ptSSR markers are highlighted in red and nSSR markers in green.

Pairs of Loci		*p*-Values	
		Caatinga	Pampa
Smolle03	Smolle10	0.02184	0.02184
Smolle08	Smolle12	0.00356	0.00356
Smolle05	Smolle19	0.00111	0.00111
Smolle14	Smolle19	0.00000	0.00000
Smolle07	Smolle21	0.03328	0.03328
Smolle19	Smolle21	0.02626	0.02626
Smolle22	Smolle17	0.03454	0.03454
Smolle07	Smolle24	0.01180	0.01180
Smolle03	Smolle25	0.00057	0.00057
Smolle11	Smolle25	0.04062	0.04062
Smolle19	Smolle27	0.00000	0.00000

## Supplementary Materials

The following are available online at http://www.mdpi.com/2079-7737/7/3/43/s1, Table S1: Genetic parameters estimated for *Schinus molle* based on the 24 SSR markers characterized in this study in overall populations and at population level; Table S2: Pairs of loci with statistically significant (*p* < 0.05) estimations of linkage disequilibrium (LD) in the populations Caatinga and Pampa; Table S3: Frequencies of private alleles in populations Pampa and Caatinga; File S4: Estimation of pollen-to-seed migration rate.
