# Supplementary material for: Characterization of Plastidial and Nuclear SSR Markers for Understanding Invasion Histories and Genetic Diversity of Schinus molle L
Source: Biology (Basel). 2018 Aug 10;7(3):43. doi: 10.3390/biology7030043 (PMC6163545; doi:10.3390/biology7030043)
Supplement: Supplementary file 1 [file biology-07-00043-s001.zip › biology-327118 supplementary for final/biology-327118-File S4.docx]

**File S4.** Estimation of pollen-to-seed migration rate.

Petit et al. (see reference [34]) demonstrated that a estimation of the pollen-to-seed migration ratio (*m_p_*/*m_s_*) is possible considering a strictly uniparental (maternal or paternal) inheritance of the plastidial genome, the biparental inheritance of the nuclear genomes and ignoring the follow unrealistic assumptions: (i) absence of mutation and selection; (ii) uniform number of migrants among populations, implying a lack of spatial genetic structure; (iii) equal male and female effective population sizes; and (iv) equilibrium between genetic drift and gene flow.

This estimation can be straightforwardly obtained through the equation:

$$\frac{m_{p}}{m_{s}}= \frac{\left[ \left( \frac{1}{F_{STb}}-1 \right) \right]\left( 1+F_{IS} \right)-2\left( \frac{1}{F_{STm}}-1 \right)}{\left( \frac{1}{G_{STm}}-1 \right)}$$

Where *F_STb_* and *F_STm_* are the estimates of population subdivision at nuclear (biparentally inherited) and maternally inherited markers respectively, and *F_IS_* = (*H_E_* − *H_O_*)/*H_E_* is the heterozygote deficit estimated with nuclear codominant markers. For species with paternally inherited organellar markers, Pettit et al. present a somewhat different equation.

Using the estimations of *F_STb_* (0.11), *F_STm_* (0.14) and *F_IS_* (0.21) obtained for populations Pampa and Caatinga in this study and considering a maternal inheritance of the plastidial genome, the pollen-to-seed ratio for *S. molle* equals 4.14.
