# Supplementary material for: Characterization of Plastidial and Nuclear SSR Markers for Understanding Invasion Histories and Genetic Diversity of Schinus molle L
Source: Biology (Basel). 2018 Aug 10;7(3):43. doi: 10.3390/biology7030043 (PMC6163545; doi:10.3390/biology7030043)
Supplement: Supplementary file 1 [file biology-07-00043-s001.zip › biology-327118 supplementary for final/biology-327118-Table S1.docx]

**Table S1.** Genetic parameters estimated for *Schinus molle* based on 25 SSR markers characterized in this study overall populations and at population level. Estimations include the number of samples (*N*), number of allele per locus (*A*), effective allele number (*A_e_*), observed (*H_O_*) and expected (*H_E_*) heterozigosities, within population fixation index (*F_IS_*) and statistical significance of the deviation from Hardy-Weinberg equilibrium (HWE).

| **Population** | **Locus** | **N** | **A** | **Ae** | **HO** | **HE** | **FIS** | **HWE** |
| --- | --- | --- | --- | --- | --- | --- | --- | --- |
| **Overall** | Smolle03 | 50.00 | 6.00 | 2.53 | 0.44 | 0.60 | 0.27 | *** |
|  | Smolle04 | 56.00 | 12.00 | 7.62 | 0.96 | 0.87 | -0.11 | *** |
|  | Smolle05 | 54.00 | 10.00 | 4.30 | 0.15 | 0.77 | 0.81 | *** |
|  | Smolle06 | 54.00 | 7.00 | 5.75 | 0.94 | 0.83 | -0.14 | *** |
|  | Smolle07 | 56.00 | 8.00 | 5.00 | 0.98 | 0.80 | -0.23 | *** |
|  | Smolle08 | 54.00 | 15.00 | 9.47 | 0.37 | 0.89 | 0.59 | *** |
|  | Smolle09 | 54.00 | 6.00 | 3.50 | 0.30 | 0.71 | 0.59 | *** |
|  | Smolle10 | 35.00 | 7.00 | 4.79 | 0.09 | 0.79 | 0.89 | *** |
|  | Smolle11 | 52.00 | 8.00 | 5.70 | 0.96 | 0.82 | -0.17 | *** |
|  | Smolle12 | 51.00 | 10.00 | 5.95 | 0.84 | 0.83 | -0.01 | *** |
|  | Smolle13 | 53.00 | 7.00 | 4.29 | 0.58 | 0.77 | 0.24 | *** |
|  | Smolle14 | 47.00 | 18.00 | 8.75 | 0.91 | 0.89 | -0.03 | *** |
|  | Smolle15 | 35.00 | 6.00 | 4.49 | 0.00 | 0.78 | 1.00 | *** |
|  | Smolle16 | 49.00 | 10.00 | 6.26 | 0.80 | 0.84 | 0.05 | *** |
|  | Smolle17 | 34.00 | 7.00 | 3.18 | 0.18 | 0.69 | 0.74 | *** |
|  | Smolle18 | 50.00 | 15.00 | 9.94 | 0.96 | 0.90 | -0.07 | *** |
|  | Smolle19 | 53.00 | 9.00 | 6.66 | 0.98 | 0.85 | -0.15 | *** |
|  | Smolle21 | 54.00 | 8.00 | 7.17 | 1.00 | 0.86 | -0.16 | *** |
|  | Smolle22 | 48.00 | 22.00 | 7.28 | 0.56 | 0.86 | 0.35 | *** |
|  | Smolle23 | 43.00 | 6.00 | 4.50 | 0.53 | 0.78 | 0.31 | *** |
|  | Smolle24 | 41.00 | 10.00 | 6.16 | 0.41 | 0.84 | 0.50 | *** |
|  | Smolle25 | 41.00 | 9.00 | 6.74 | 0.71 | 0.85 | 0.17 | *** |
|  | Smolle27 | 48.00 | 18.00 | 10.57 | 0.98 | 0.91 | -0.08 | *** |
|  | Smolle28 | 49.00 | 8.00 | 4.15 | 0.86 | 0.76 | -0.13 | *** |
|  | Smolle30 | 53.00 | 29.00 | 13.47 | 0.91 | 0.93 | 0.02 | *** |
|  | Mean | 48.56 | 10.84 | 6.33 | 0.66 | 0.82 | 0.21 | - |
| **Caatinga** | Smolle03 | 25 | 2.00 | 1.57 | 0.00 | 0.36 | 1.00 | *** |
|  | Smolle04 | 29 | 10.00 | 6.95 | 1.00 | 0.86 | -0.17 | *** |
|  | Smolle05 | 29 | 5.00 | 3.52 | 0.00 | 0.72 | 1.00 | *** |
|  | Smolle06 | 29 | 4.00 | 3.84 | 0.90 | 0.74 | -0.21 | *** |
|  | Smolle07 | 29 | 6.00 | 3.36 | 1.00 | 0.70 | -0.42 | *** |
|  | Smolle08 | 29 | 12.00 | 8.41 | 0.45 | 0.88 | 0.49 | *** |
|  | Smolle09 | 29 | 2.00 | 1.58 | 0.00 | 0.37 | 1.00 | *** |
|  | Smolle10 | 13 | 3.00 | 2.00 | 0.08 | 0.50 | 0.85 | ** |
|  | Smolle11 | 26 | 5.00 | 3.53 | 1.00 | 0.72 | -0.40 | *** |
|  | Smolle12 | 26 | 7.00 | 4.55 | 0.88 | 0.78 | -0.13 | *** |
|  | Smolle13 | 26 | 4.00 | 1.93 | 0.15 | 0.48 | 0.68 | *** |
|  | Smolle14 | 23 | 11.00 | 5.81 | 0.91 | 0.83 | -0.10 | *** |
|  | Smolle15 | 11 | 3.00 | 2.12 | 0.00 | 0.53 | 1.00 | *** |
|  | Smolle16 | 25 | 10.00 | 7.06 | 0.76 | 0.86 | 0.11 | ** |
|  | Smolle17 | 16 | 6.00 | 3.76 | 0.00 | 0.73 | 1.00 | *** |
|  | Smolle18 | 25 | 11.00 | 7.35 | 0.96 | 0.86 | -0.11 | *** |
|  | Smolle19 | 28 | 4.00 | 3.99 | 1.00 | 0.75 | -0.33 | *** |
|  | Smolle21 | 29 | 6.00 | 5.68 | 1.00 | 0.82 | -0.21 | *** |
|  | Smolle22 | 28 | 20.00 | 15.84 | 0.96 | 0.94 | -0.03 | * |
|  | Smolle23 | 27 | 5.00 | 4.84 | 0.85 | 0.79 | -0.07 | *** |
|  | Smolle24 | 23 | 9.00 | 6.70 | 0.74 | 0.85 | 0.13 | *** |
|  | Smolle25 | 21 | 8.00 | 4.37 | 0.71 | 0.77 | 0.07 | *** |
|  | Smolle27 | 25 | 17.00 | 11.57 | 1.00 | 0.91 | -0.09 | *** |
|  | Smolle28 | 23 | 6.00 | 3.40 | 0.74 | 0.71 | -0.05 | ** |
|  | Smolle30 | 29 | 26.00 | 14.88 | 1.00 | 0.93 | -0.07 | *** |
|  | Mean | 24.920 | 8.08 | 5.55 | 0.64 | 0.74 | 0.20 | - |
| **Pampa** | Smolle03 | 25 | 6.00 | 3.69 | 0.88 | 0.73 | -0.21 | *** |
|  | Smolle04 | 27 | 8.00 | 4.40 | 0.93 | 0.77 | -0.20 | *** |
|  | Smolle05 | 25 | 10.00 | 4.88 | 0.32 | 0.80 | 0.60 | *** |
|  | Smolle06 | 25 | 4.00 | 3.01 | 1.00 | 0.67 | -0.50 | *** |
|  | Smolle07 | 27 | 5.00 | 3.12 | 0.96 | 0.68 | -0.42 | *** |
|  | Smolle08 | 25 | 6.00 | 3.48 | 0.28 | 0.71 | 0.61 | *** |
|  | Smolle09 | 25 | 5.00 | 3.32 | 0.64 | 0.70 | 0.08 | ns |
|  | Smolle10 | 22 | 5.00 | 3.00 | 0.09 | 0.67 | 0.86 | *** |
|  | Smolle11 | 26 | 5.00 | 3.82 | 0.92 | 0.74 | -0.25 | *** |
|  | Smolle12 | 25 | 7.00 | 5.14 | 0.80 | 0.81 | 0.01 | *** |
|  | Smolle13 | 27 | 4.00 | 2.46 | 1.00 | 0.59 | -0.69 | *** |
|  | Smolle14 | 24 | 9.00 | 4.48 | 0.92 | 0.78 | -0.18 | *** |
|  | Smolle15 | 24 | 4.00 | 2.72 | 0.00 | 0.63 | 1.00 | *** |
|  | Smolle16 | 24 | 6.00 | 3.03 | 0.83 | 0.67 | -0.24 | *** |
|  | Smolle17 | 18 | 5.00 | 1.72 | 0.33 | 0.42 | 0.21 | *** |
|  | Smolle18 | 25 | 7.00 | 4.92 | 0.96 | 0.80 | -0.20 | ** |
|  | Smolle19 | 25 | 7.00 | 6.31 | 0.96 | 0.84 | -0.14 | *** |
|  | Smolle21 | 25 | 5.00 | 3.71 | 1.00 | 0.73 | -0.37 | *** |
|  | Smolle22 | 20 | 3.00 | 1.50 | 0.00 | 0.34 | 1.00 | *** |
|  | Smolle23 | 16 | 3.00 | 2.33 | 0.00 | 0.57 | 1.00 | *** |
|  | Smolle24 | 18 | 3.00 | 2.42 | 0.00 | 0.59 | 1.00 | *** |
|  | Smolle25 | 20 | 5.00 | 3.79 | 0.70 | 0.74 | 0.05 | ns |
|  | Smolle27 | 23 | 8.00 | 6.15 | 0.96 | 0.84 | -0.14 | *** |
|  | Smolle28 | 26 | 3.00 | 2.14 | 0.96 | 0.53 | -0.80 | *** |
|  | Smolle30 | 24 | 6.00 | 4.25 | 0.79 | 0.76 | -0.04 | *** |
|  | Mean | 23.640 | 5.56 | 3.59 | 0.65 | 0.68 | 0.08 | - |

Statistical significance: *** = *p* < 0.001; ** = *p* < 0.01; * = *p* < 0.05; ns: not significant.
