# Supplementary material for: Characterization of Plastidial and Nuclear SSR Markers for Understanding Invasion Histories and Genetic Diversity of Schinus molle L
Source: Biology (Basel). 2018 Aug 10;7(3):43. doi: 10.3390/biology7030043 (PMC6163545; doi:10.3390/biology7030043)
Supplement: Supplementary file 1 [file biology-07-00043-s001.zip › biology-327118 supplementary for final/biology-327118-Table S2.docx]

**Table S2.** Pairs of loci with statistically significant (*p* < 0.05) estimations of linkage disequilibrium (LD) in populations Caatinga and Pampa. The 12 first pairs of loci presented significant LD for both populations.

| **Caatinga** | | | **Pampa** | | |
| --- | --- | --- | --- | --- | --- |
| **Pairs of loci** | | ***p*-Value** | **Pairs of loci** | | ***p*-Value** |
| **Smolle03** | **Smolle10** | **0.033810** | **Smolle03** | **Smolle10** | **0.021840** |
| **Smolle08** | **Smolle12** | **0.010840** | **Smolle08** | **Smolle12** | **0.003560** |
| **Smolle05** | **Smolle19** | **0.014700** | **Smolle05** | **Smolle19** | **0.001110** |
| **Smolle14** | **Smolle19** | **0.029360** | **Smolle14** | **Smolle19** | **0.000000** |
| **Smolle07** | **Smolle21** | **0.016840** | **Smolle07** | **Smolle21** | **0.033280** |
| **Smolle19** | **Smolle21** | **0.035270** | **Smolle19** | **Smolle21** | **0.026260** |
| **Smolle17** | **Smolle22** | **0.013540** | **Smolle17** | **Smolle22** | **0.034540** |
| **Smolle07** | **Smolle24** | **0.047930** | **Smolle07** | **Smolle24** | **0.011800** |
| **Smolle03** | **Smolle25** | **0.037240** | **Smolle03** | **Smolle25** | **0.000570** |
| **Smolle11** | **Smolle25** | **0.042020** | **Smolle11** | **Smolle25** | **0.040620** |
| **Smolle19** | **Smolle27** | **0.031900** | **Smolle19** | **Smolle27** | **0.000000** |
| **Smolle05** | **Smolle30** | **0.013130** | **Smolle05** | **Smolle30** | **0.026600** |
| Smolle03 | Smolle07 | 0.001160 | Smolle03 | Smolle05 | 0.000050 |
| Smolle04 | Smolle07 | 0.036780 | Smolle07 | Smolle08 | 0.032690 |
| Smolle04 | Smolle09 | 0.034040 | Smolle05 | Smolle12 | 0.027990 |
| Smolle08 | Smolle09 | 0.037900 | Smolle10 | Smolle12 | 0.036100 |
| Smolle05 | Smolle10 | 0.022280 | Smolle03 | Smolle13 | 0.000090 |
| Smolle07 | Smolle10 | 0.022090 | Smolle05 | Smolle13 | 0.001520 |
| Smolle04 | Smolle12 | 0.000220 | Smolle07 | Smolle13 | 0.019330 |
| Smolle07 | Smolle12 | 0.006810 | Smolle08 | Smolle14 | 0.000920 |
| Smolle04 | Smolle14 | 0.000000 | Smolle11 | Smolle14 | 0.008690 |
| Smolle12 | Smolle14 | 0.000000 | Smolle10 | Smolle15 | 0.048180 |
| Smolle03 | Smolle15 | 0.034340 | Smolle11 | Smolle15 | 0.000000 |
| Smolle07 | Smolle15 | 0.008120 | Smolle03 | Smolle16 | 0.006690 |
| Smolle09 | Smolle15 | 0.036130 | Smolle05 | Smolle16 | 0.004140 |
| Smolle12 | Smolle15 | 0.040690 | Smolle06 | Smolle16 | 0.029980 |
| Smolle09 | Smolle16 | 0.005760 | Smolle07 | Smolle16 | 0.015570 |
| Smolle10 | Smolle16 | 0.012570 | Smolle08 | Smolle16 | 0.000000 |
| Smolle11 | Smolle16 | 0.044060 | Smolle12 | Smolle16 | 0.018000 |
| Smolle03 | Smolle17 | 0.010300 | Smolle13 | Smolle16 | 0.000440 |
| Smolle06 | Smolle17 | 0.024060 | Smolle08 | Smolle17 | 0.013710 |
| Smolle07 | Smolle17 | 0.044510 | Smolle12 | Smolle17 | 0.045640 |
| Smolle09 | Smolle17 | 0.022860 | Smolle08 | Smolle19 | 0.024620 |
| Smolle10 | Smolle17 | 0.037310 | Smolle12 | Smolle19 | 0.011790 |
| Smolle11 | Smolle17 | 0.013950 | Smolle13 | Smolle19 | 0.031730 |
| Smolle16 | Smolle17 | 0.014410 | Smolle05 | Smolle21 | 0.043380 |
| Smolle05 | Smolle18 | 0.018290 | Smolle13 | Smolle21 | 0.028750 |
| Smolle06 | Smolle18 | 0.003310 | Smolle18 | Smolle22 | 0.028400 |
| Smolle07 | Smolle18 | 0.043350 | Smolle14 | Smolle23 | 0.035840 |
| Smolle10 | Smolle18 | 0.040430 | Smolle19 | Smolle23 | 0.031310 |
| Smolle12 | Smolle18 | 0.026900 | Smolle03 | Smolle24 | 0.000580 |
| Smolle13 | Smolle18 | 0.061090 | Smolle05 | Smolle24 | 0.021360 |
| Smolle15 | Smolle18 | 0.026320 | Smolle08 | Smolle24 | 0.017670 |
| Smolle17 | Smolle18 | 0.002370 | Smolle13 | Smolle24 | 0.000790 |
| Smolle06 | Smolle19 | 0.028980 | Smolle14 | Smolle24 | 0.012680 |
| Smolle08 | Smolle21 | 0.031390 | Smolle16 | Smolle24 | 0.001710 |
| Smolle12 | Smolle21 | 0.014490 | Smolle19 | Smolle24 | 0.028450 |
| Smolle17 | Smolle21 | 0.034840 | Smolle04 | Smolle25 | 0.011490 |
| Smolle18 | Smolle21 | 0.000000 | Smolle14 | Smolle25 | 0.007370 |
| Smolle05 | Smolle22 | 0.007160 | Smolle16 | Smolle25 | 0.011080 |
| Smolle09 | Smolle22 | 0.021460 | Smolle19 | Smolle25 | 0.020560 |
| Smolle16 | Smolle22 | 0.018470 | Smolle12 | Smolle27 | 0.021980 |
| Smolle05 | Smolle23 | 0.001890 | Smolle13 | Smolle27 | 0.023890 |
| Smolle08 | Smolle23 | 0.033050 | Smolle14 | Smolle27 | 0.046660 |
| Smolle10 | Smolle23 | 0.001850 | Smolle21 | Smolle27 | 0.017560 |
| Smolle12 | Smolle23 | 0.000520 | Smolle03 | Smolle28 | 0.029470 |
| Smolle15 | Smolle23 | 0.026350 | Smolle03 | Smolle30 | 0.034830 |
| Smolle17 | Smolle23 | 0.022600 | Smolle13 | Smolle30 | 0.030720 |
| Smolle18 | Smolle23 | 0.002540 | Smolle16 | Smolle30 | 0.048910 |
| Smolle21 | Smolle23 | 0.033110 | - | - | - |
| Smolle11 | Smolle24 | 0.013770 | - | - | - |
| Smolle17 | Smolle24 | 0.030490 | - | - | - |
| Smolle18 | Smolle24 | 0.001020 | - | - | - |
| Smolle21 | Smolle24 | 0.002400 | - | - | - |
| Smolle23 | Smolle24 | 0.005180 | - | - | - |
| Smolle05 | Smolle25 | 0.006620 | - | - | - |
| Smolle10 | Smolle25 | 0.011370 | - | - | - |
| Smolle23 | Smolle25 | 0.012360 | - | - | - |
| Smolle15 | Smolle27 | 0.047790 | - | - | - |
| Smolle18 | Smolle27 | 0.011030 | - | - | - |
| Smolle10 | Smolle28 | 0.048300 | - | - | - |
| Smolle16 | Smolle28 | 0.038200 | - | - | - |
| Smolle08 | Smolle30 | 0.013800 | - | - | - |
| Smolle12 | Smolle30 | 0.018550 | - | - | - |
| Smolle17 | Smolle30 | 0.038560 | - | - | - |
| Smolle21 | Smolle30 | 0.009650 | - | - | - |
| Smolle22 | Smolle30 | 0.033110 | - | - | - |
