# Supplementary material for: Characterization of Plastidial and Nuclear SSR Markers for Understanding Invasion Histories and Genetic Diversity of Schinus molle L
Source: Biology (Basel). 2018 Aug 10;7(3):43. doi: 10.3390/biology7030043 (PMC6163545; doi:10.3390/biology7030043)
Supplement: Supplementary file 1 [file biology-07-00043-s001.zip › biology-327118 supplementary for final/biology-327118-Table S3.docx]

**Table S3.** Frequencies of private alleles in populations Pampa (highlighted in yellow) and Caatinga (highlighted in green).

| **Locus** | **Allele** | **Caatinga** | **Pampa** |
| --- | --- | --- | --- |
| **ptSSR markers** | | | |
| **Smolle03** |  |  |  |
|  | **133** | 0.000 | 0.060 |
|  | **190** | 0.000 | 0.160 |
|  | **193** | 0.000 | 0.240 |
|  | **196** | 0.000 | 0.040 |
| **Smolle04** |  |  |  |
|  | **133** | 0.017 | 0.000 |
|  | **219** | 0.000 | 0.037 |
|  | **221** | 0.000 | 0.241 |
|  | **227** | 0.155 | 0.000 |
|  | **229** | 0.172 | 0.000 |
|  | **231** | 0.086 | 0.000 |
| **Smolle05** |  |  |  |
|  | **292** | 0.000 | 0.040 |
|  | **298** | 0.000 | 0.040 |
|  | **301** | 0.000 | 0.020 |
|  | **304** | 0.000 | 0.020 |
|  | **307** | 0.000 | 0.080 |
| **Smolle07** |  |  |  |
|  | **106** | 0.000 | 0.019 |
|  | **115** | 0.052 | 0.000 |
|  | **133** | 0.000 | 0.167 |
|  | **139** | 0.362 | 0.000 |
|  | **142** | 0.017 | 0.000 |
| **Smolle09** |  |  |  |
|  | **208** | 0.759 | 0.000 |
|  | **214** | 0.000 | 0.360 |
|  | **223** | 0.000 | 0.040 |
|  | **226** | 0.000 | 0.260 |
|  | **229** | 0.000 | 0.020 |
| **Smolle10** |  |  |  |
|  | **250** | 0.615 | 0.000 |
|  | **253** | 0.346 | 0.000 |
|  | **256** | 0.000 | 0.227 |
|  | **262** | 0.000 | 0.273 |
|  | **268** | 0.000 | 0.023 |
|  | **271** | 0.000 | 0.023 |
| **Smolle12** |  |  |  |
|  | **118** | 0.000 | 0.120 |
|  | **121** | 0.000 | 0.120 |
|  | **124** | 0.000 | 0.160 |
|  | **136** | 0.019 | 0.000 |
|  | **139** | 0.115 | 0.000 |
|  | **142** | 0.288 | 0.000 |
| **Smolle13** |  |  |  |
|  | **115** | 0.000 | 0.481 |
|  | **117** | 0.000 | 0.019 |
|  | **139** | 0.250 | 0.000 |
|  | **141** | 0.673 | 0.000 |
|  | **167** | 0.019 | 0.000 |
|  | **169** | 0.000 | 0.407 |
| **Smolle14** |  |  |  |
|  | **121** | 0.000 | 0.208 |
|  | **123** | 0.000 | 0.146 |
|  | **127** | 0.000 | 0.021 |
|  | **129** | 0.000 | 0.042 |
|  | **133** | 0.000 | 0.021 |
|  | **157** | 0.022 | 0.000 |
|  | **159** | 0.087 | 0.000 |
|  | **161** | 0.065 | 0.000 |
|  | **163** | 0.261 | 0.000 |
|  | **165** | 0.022 | 0.000 |
|  | **177** | 0.000 | 0.063 |
|  | **179** | 0.043 | 0.000 |
|  | **185** | 0.261 | 0.000 |
|  | **187** | 0.043 | 0.000 |
|  | **191** | 0.022 | 0.000 |
| **Smolle19** |  |  |  |
|  | **123** | 0.000 | 0.160 |
|  | **125** | 0.000 | 0.180 |
|  | **127** | 0.000 | 0.140 |
|  | **149** | 0.232 | 0.000 |
|  | **151** | 0.268 | 0.000 |
| **Smolle22** |  |  |  |
|  | **157** | 0.018 | 0.000 |
|  | **159** | 0.036 | 0.000 |
|  | **161** | 0.036 | 0.000 |
|  | **163** | 0.054 | 0.000 |
|  | **165** | 0.071 | 0.000 |
|  | **167** | 0.054 | 0.000 |
|  | **169** | 0.054 | 0.000 |
|  | **171** | 0.125 | 0.000 |
|  | **173** | 0.036 | 0.000 |
|  | **187** | 0.000 | 0.150 |
|  | **189** | 0.000 | 0.800 |
|  | **193** | 0.054 | 0.000 |
|  | **195** | 0.018 | 0.000 |
|  | **197** | 0.036 | 0.000 |
|  | **199** | 0.036 | 0.000 |
|  | **201** | 0.054 | 0.000 |
|  | **203** | 0.036 | 0.000 |
|  | **205** | 0.071 | 0.000 |
|  | **207** | 0.071 | 0.000 |
|  | **209** | 0.089 | 0.000 |
|  | **211** | 0.036 | 0.000 |
| **EST-SSR markers** | | | |
| **Smolle06** |  |  |  |
|  | **115** | 0.000 | 0.060 |
|  | **117** | 0.000 | 0.440 |
|  | **143** | 0.276 | 0.000 |
|  | **145** | 0.172 | 0.000 |
|  | **159** | 0.241 | 0.000 |
|  | **161** | 0.000 | 0.320 |
| **Smolle08** |  |  |  |
|  | **289** | 0.000 | 0.460 |
|  | **291** | 0.000 | 0.180 |
|  | **293** | 0.000 | 0.060 |
|  | **303** | 0.138 | 0.000 |
|  | **305** | 0.138 | 0.000 |
|  | **307** | 0.207 | 0.000 |
|  | **309** | 0.086 | 0.000 |
|  | **311** | 0.034 | 0.000 |
|  | **313** | 0.086 | 0.000 |
|  | **315** | 0.034 | 0.000 |
|  | **317** | 0.069 | 0.000 |
|  | **319** | 0.017 | 0.000 |
| **Smolle11** |  |  |  |
|  | **118** | 0.000 | 0.231 |
|  | **121** | 0.000 | 0.212 |
|  | **124** | 0.000 | 0.019 |
|  | **145** | 0.385 | 0.000 |
|  | **148** | 0.038 | 0.000 |
|  | **151** | 0.212 | 0.000 |
| **Smolle15** |  |  |  |
|  | **283** | 0.000 | 0.125 |
|  | **286** | 0.000 | 0.375 |
|  | **289** | 0.000 | 0.458 |
|  | **295** | 0.182 | 0.000 |
|  | **298** | 0.636 | 0.000 |
| **Smolle28** |  |  |  |
|  | **109** | 0.000 | 0.442 |
|  | **111** | 0.000 | 0.038 |
|  | **127** | 0.043 | 0.000 |
|  | **129** | 0.261 | 0.000 |
|  | **131** | 0.065 | 0.000 |
|  | **143** | 0.435 | 0.000 |
|  | **145** | 0.022 | 0.000 |
| **Smolle30** |  |  |  |
|  | **121** | 0.000 | 0.042 |
|  | **123** | 0.000 | 0.167 |
|  | **125** | 0.000 | 0.188 |
|  | **149** | 0.017 | 0.000 |
|  | **153** | 0.017 | 0.000 |
|  | **155** | 0.034 | 0.000 |
|  | **157** | 0.017 | 0.000 |
|  | **159** | 0.017 | 0.000 |
|  | **163** | 0.017 | 0.000 |
|  | **165** | 0.017 | 0.000 |
|  | **167** | 0.017 | 0.000 |
|  | **175** | 0.052 | 0.000 |
|  | **177** | 0.103 | 0.000 |
|  | **179** | 0.138 | 0.000 |
|  | **181** | 0.052 | 0.000 |
|  | **183** | 0.017 | 0.000 |
|  | **187** | 0.017 | 0.000 |
|  | **189** | 0.017 | 0.000 |
|  | **193** | 0.052 | 0.000 |
|  | **195** | 0.017 | 0.000 |
|  | **197** | 0.017 | 0.000 |
|  | **199** | 0.034 | 0.000 |
|  | **201** | 0.052 | 0.000 |
|  | **203** | 0.052 | 0.000 |
|  | **205** | 0.121 | 0.000 |
|  | **209** | 0.017 | 0.000 |

| **nSSR markers** | | | |
| --- | --- | --- | --- |
| **Smolle17** |  |  |  |
|  | **211** | 0.125 | 0.000 |
|  | **214** | 0.063 | 0.000 |
|  | **229** | 0.000 | 0.083 |
| **Smolle18** |  |  |  |
|  | **109** | 0.000 | 0.020 |
|  | **111** | 0.000 | 0.140 |
|  | **113** | 0.000 | 0.240 |
|  | **115** | 0.000 | 0.080 |
|  | **129** | 0.100 | 0.000 |
|  | **131** | 0.040 | 0.000 |
|  | **133** | 0.100 | 0.000 |
|  | **135** | 0.220 | 0.000 |
|  | **137** | 0.020 | 0.000 |
|  | **151** | 0.200 | 0.000 |
|  | **153** | 0.100 | 0.000 |
|  | **155** | 0.020 | 0.000 |
| **Smolle21** |  |  |  |
|  | **105** | 0.000 | 0.160 |
|  | **107** | 0.000 | 0.340 |
|  | **125** | 0.155 | 0.000 |
|  | **127** | 0.224 | 0.000 |
|  | **135** | 0.172 | 0.000 |
| **Smolle23** |  |  |  |
|  | **175** | 0.167 | 0.000 |
|  | **178** | 0.259 | 0.000 |
|  | **217** | 0.000 | 0.125 |
|  | **226** | 0.167 | 0.000 |
| **Smolle24** |  |  |  |
|  | **184** | 0.109 | 0.000 |
|  | **187** | 0.174 | 0.000 |
|  | **190** | 0.065 | 0.000 |
|  | **193** | 0.022 | 0.000 |
|  | **244** | 0.000 | 0.556 |
|  | **253** | 0.174 | 0.000 |
|  | **256** | 0.087 | 0.000 |
|  | **259** | 0.022 | 0.000 |
| **Smolle25** |  |  |  |
|  | **118** | 0.000 | 0.125 |
|  | **127** | 0.167 | 0.000 |
|  | **130** | 0.095 | 0.000 |
|  | **169** | 0.095 | 0.000 |
|  | **172** | 0.405 | 0.000 |
| **Smolle27** |  |  |  |
|  | **181** | 0.020 | 0.000 |
|  | **183** | 0.020 | 0.000 |
|  | **185** | 0.100 | 0.000 |
|  | **187** | 0.120 | 0.000 |
|  | **189** | 0.120 | 0.000 |
|  | **191** | 0.080 | 0.000 |
|  | **195** | 0.020 | 0.000 |
|  | **197** | 0.020 | 0.000 |
|  | **257** | 0.000 | 0.022 |
|  | **259** | 0.020 | 0.000 |
|  | **261** | 0.020 | 0.000 |
